# Ghana beyond the epi-curve: initial lessons learned from the implementation of infection prevention and control measures in the COVID-19 response

**DOI:** 10.11604/pamj.2021.38.18.26832

**Published:** 2021-01-07

**Authors:** Mary Eyram Ashinyo

**Affiliations:** 1 Department of Quality Assurance, Institutional Care Division, Ghana Health Service Headquarters, Private Mail bag, Accra, Ghana

**Keywords:** Safety, infectious disease, infection prevention and control, Ghana, COVID-19

## Abstract

Infection prevention and control (IPC) measures remain crucial to breaking transmission of the virus in the wake of inconclusive efforts underway to find an effective vaccine and treatment. While acknowledging that many lessons evolve as the pandemic unfolds, an initial understanding and recognition of the complexities that surround IPC policy implementation and adherence is vital for effective control of on-going pandemic in particular and to inform national IPC policies beyond the epi-curve. This short communication therefore seeks to unravel initial thoughts, themes and concepts that have unfolded in the implementation of IPC policies and guidelines in the context of the ongoing outbreak response in Ghana. A rapid desk review was done. Reflexive journals, field notes, observations and workshop experiences were compiled and overlapped with authors’ experience as a member of the COVID-19 national response team for Infection Prevention and Control (IPC). Thematic content analysis was then used to categorize the lessons into common themes. While aligning with global strategies, the concept of ‘looking within’ for initial solutions and strengths have proven useful for a public health emergency response in Ghana. Future IPC policies must inculcate perspectives from the politics and economics of IPC practices and employ varieties of multidisciplinary approaches required to broaden the scope of IPC practice.

## Introduction

The corona virus disease 2019 (COVID-19) is now a pandemic [1-3]. It has been devastating to the world with over 40 million cases and 1.1 million deaths reported globally as at October 18 2020. Africa has relatively less disease burden with only 3 percent of the confirmed cases and 3 percent of the deaths reported globally [4]. In Ghana, over forty-seven thousand confirmed cases had been reported with three hundred and ten deaths as at 21^st^ October 2020 [5]. The overall aim of the global pandemic response is to keep COVID-19 under control by suppressing viral transmission and thereby reducing diseases and death from the virus [6]. Appropriate Infection Prevention and Control (IPC) measures ‘such as hand hygiene with soap and water, and the use of hand sanitizer containing ≥60% alcohol, wearing of face mask, social distancing , as well as the use of appropriate personal protective equipment are recommended to prevent infection with SARS-CoV-2 which causes COVID-19´ [7]. Similarly, Ghana´s preventive measures included: (1) city lockdown of two epi centres and closure of public places, (2) compulsory quarantine and testing of travelers, (3) early detection and isolation of infected persons through temperature screening, contact tracing and enhanced surveillance, (4) practice of social distancing and hand hygiene and (5) the use of risk-based Personal Protective Equipment [5]. An initial understanding, documentation and recognition of the complexities that surround IPC policy implementation and adherence are important for effective control of on-going pandemic in particular and infectious diseases in general. This short communication therefore seeks to unravel initial thoughts, themes and concepts that have unfolded in the implementation of IPC policies and guidelines in the context of the ongoing outbreak response in Ghana.

## Methods

A rapid desk review was done. Reflexive journals, field notes, observations and workshop experiences were compiled and overlapped with author´s experience as a member of the COVID-19 national response team for infection prevention and control. Thematic content analysis was then used to categorize the findings into common emerging themes.

## Results

Initial lessons learned have emerged around four main themes namely; (1) human resources development, (2) governance and scope, (3) information management and research and (4) the politics, economics and ethics of preventive and control measures. While it is important to ensure that country mechanisms are evidence based and aligned with international mechanisms, the concept of 'looking within' has emerged strongly as a key lesson learned across all thematic areas for implementing preventive and control measures in Ghana. The concept of 'looking within' is generally understood in Ghana's context as harnessing and mobilizing material, monetary and man-power resources within country's existing public health and administrative systems and structures as initial and continuing contextual evidence-based interventions without over-relying on external strategies, solutions, standards and aid. Ghana has demonstrated this across the four themes by mobilizing its existing public health workforce and its experience with the support rendered during the Ebola virus response to affected peers, activating and enhancing existing public health surveillance, response structures and systems, enhancing existing reporting and command systems and promoting self-reliance through the empowerment of local standardization and production of IPC logistics such as hand sanitizers and PPEs.

## Discussion

**Human resources development for IPC:** a quick human resource needs assessment and existing capacity has been useful in Ghana. A key lesson learned is that knowledge and practice of IPC are required across all specialties of the public health workforce and not only the clinical professionals. The ability to acquire such technical capacity can be constrained by healthcare professionals' degree of education. It has therefore proven useful to design separate modules for clinical and non-clinical staff as well as translate teaching content to local languages that are understood by cadres who have lower educational levels where need be, such as mortuary workers. Additionally, inadequate IPC preparedness increased health worker anxiety about their safety and diminishes their confidence to provide safe care. Regular risk assessment of healthcare workers therefore helped to institute the appropriate strategies for health worker safety particularly in reducing infections among them. Also, remote learning and guidelines dissemination using webinars, communities of practice presented new opportunities for IPC capacity building however, health workers found remote learning and training activities more useful where these were accompanied with appropriate supportive supervisory strategies to support their continual learning and performance.

**Infection prevention and control governance and scope:** evidence-based strategies, guidelines, clear policies and communication structures are important for IPC practice decision making. A quick review to identify strengths and gaps in existing IPC policies and guidelines have been useful. It has become more emphasized that decision making around IPC implementation requires strong leadership and political commitment at all levels. Such leadership support may include resource mobilization and allocation for program funding, capacity building activities, provision of hand hygiene logistics and personal protective equipment, discussion of IPC issues at high-level meeting and review meetings, invitation of IPC stakeholders to meetings and strong monitoring and evaluation systems for IPC programs. Integrating IPC guidelines into existing programs while standardizing IPC tools and adapting these guidelines to local context have been helpful. Rapid but effective local accreditation and regulatory mechanisms to assure safe production of IPC logistics including PPEs and hand hygiene products were key strategies to meet country's demand. Recognizing power relationships in the IPC decision process enabled appropriate negotiations among stakeholders. Additionally, the scope of IPC preparedness must be broadened to include communities such as prisons, work places and corporate settings, schools, markets and refugee camps early enough in the response strategies. Finally, IPC governance in Ghana requires strong partnerships with diverse stakeholders for effective implementation.

**Infection prevention and control information management and research:** the COVID-19 response in Ghana has highlighted the need to look within health systems for influential local leaders who are able to build trust and social capital with communities for effective communication and collective IPC compliance. Such communication must be timely, audience and context appropriate and sustained through out the response. Similarly, while Ghana drew content from global health authorities, policy makers also looked within to generate local evidence from Ghanaian researchers to inform key policies.

**The politics, economics and ethics of preventive and control measures:** health economists generally argue that health as a public good does not obey open market principles, the pandemic has however highlighted the huge economics of demand and supply forces that lace the supply chain of IPC logistics, how these influence IPC policy formulation and implementation, and ethical issues that arise therein. Governments can mitigate these open market forces by public-private partnerships to keep the cost of these logistics in acceptable limits within which they remain safe, affordable and available to healthcare workers and the general population. Similarly, non-direct IPC logistics such as testing systems are prone to subtle open market forces thus availability of such logistics may also drive IPC policy formulation and implementation. For instance, this has been seen in the advocacy for IPC logistics such as mask to be reserved for the use of health workers at the early stage of the response since they were at higher risk of exposure to the virus. Ethical issues thus arise under such conditions when masks should have otherwise been used, as well as when health workers need to attend to confirmed infectious cases without adequate PPEs. Ghana controlled these open market forces through an intentional self-reliance strategy by looking within for local production of IPC logistics.

## Conclusion

The 'looking within' approach has proven useful in IPC governance, human resource capacity building, logistics supply and IPC education in Ghana's COVID-19 response. A variety of multidisciplinary approaches are required with a focus across clinical, public health and administrative domains. Local infrastructure and systems for IPC have proven useful in responding to a pandemic such as the on-going COVID-19. Additional lessons learned need to be institutionalized into existing IPC policy formation and implementation for sustainability and continuous IPC learning for routine IPC practice and future outbreak responses. Future IPC policies must inculcate perspectives from the politics and economics of IPC practices and employ a variety of multidisciplinary approaches required to broaden the scope of IPC programs.

### What is known about this topic

- The corona virus disease 2019 (COVID-19) is now a pandemic and has been devastating to the world;
- Infection prevention and control measures remain crucial to breaking transmission of SAR-COV-2 in the wake of inconclusive therapeutic interventions.

### What this study adds

- While aligning with global policy is important, 'looking within' country's existing public health systems as initial and continuing contextual evidence-based interventions without over-relying on external strategies and solutions presents a useful approach in a public health emergency;
- The pandemic highlights open market forces that lace the supply chain of IPC logistics and how these influence IPC policy formulation and implementation.
